# Measurement Issue in Antecollis

**DOI:** 10.5334/tohm.723

**Published:** 2022-10-10

**Authors:** Kyung Ah Woo, Jae Young Joo, Jung Hwan Shin, Chan Young Lee, Seung-Ho Jeon, Jee-Young Lee, Han-Joon Kim, Beomseok Jeon

**Affiliations:** 1 Department of Neurology, Seoul National University Hospital, Seoul, South Korea; 2 Department of Neurology, School of Medicine, Ewha Womans University Mokdong Hospital, Seoul, South Korea; 3 Department of Neurology, Seoul National University College of Medicine, Seoul, South Korea; 4 Department of Neurology, Seoul Metropolitan Government-Seoul National University Boramae Medical Center, Seoul, South Korea

**Keywords:** Camptocormia, Parkinsonism, Postural balance, Bent Spine Syndrome

## Abstract

**Background:**

Antecollis is defined as an involuntary forward flexion of the neck. Previous reports have measured the neck flexion angles based on the line perpendicular to the ground. This led to an inflation of the neck flexion angles in patients who had combined forward truncal flexions, especially upper camptocormia.

**Methods:**

We examined the neck flexion angles and the upper camptocormia angle in the published photographs of antecollis. MEDLINE search was conducted using the following search terms: *antecollis* OR *anterocollis*. Lateral-view photographs of patients diagnosed with antecollis were collected. Neck flexion angles were measured with the classic ‘perpendicular method’ and the ‘antecollis method’ we developed.

**Results:**

Nine patient photographs were identified. While antecollis was the only described postural abnormality in eight cases, these patients exhibited upper camptocormia angles of 45° or larger. The mean neck flexion angle measured with the antecollis method was 49.7°, while the perpendicular method yielded 103.4°.

**Discussion:**

Upper camptocormia should be considered in the evaluation of antecollis. We propose a new method to measure neck flexion in relation to the torso, instead of the vertical line.

## Introduction

Antecollis is characterized by marked, involuntary forward flexion of the head and neck area. Although frequently associated with parkinsonian disorders, the pathophysiology of antecollis is not well understood. Observational evidence shows a higher prevalence of antecollis in multiple system atrophy (MSA) than in Parkinson’s disease (PD), contrary to camptocormia which is relatively common in PD [1]. The term ‘disproportionate antecollis’ indicates a marked forward flexion of the neck that is more pronounced than anticipated in comparison to the flexure of the trunk and is considered a ‘red flag’ sign that points to MSA over PD [2,3].

Still, there is no standardized method to assess the severity of antecollis. Several studies have measured the angle between the vertical line and the neck, but this angle is affected by truncal flexion because it uses the vertical line. Hence, the presence of camptocormia contributes to an overestimation of the neck flexion angle in this measurement. Upper camptocormia can cause more confusion in cases it is not visually distinct from antecollis. Given the difference in the prevalence of antecollis and camptocormia between PD and MSA, this calls for a new clinical approach to differentiate between these two postural abnormalities in parkinsonian patients.

Herein, we reviewed the photographs of the patients diagnosed with antecollis, and examined the postural angles of upper camptocormia and antecollis to reconsider the diagnosis. We propose a new measurement method for antecollis to reliably assess neck flexion in patients with combined truncal flexion.

## Methods

### Search of Literature

We conducted a MEDLINE search using the following search terms: *antecollis* OR *anterocollis*. All publications in English were included with no restriction for publication dates. The last search was performed on July 22^nd^, 2020.

We collected the lateral-view photographs of individual patients diagnosed with antecollis in the articles. In order to measure both the neck flexion angles and the upper camptocormia angle, the points of C7 and L5 spinous processes had to be visually estimable in the photographs. Therefore, we excluded photographs which covered only the cervical region or those taken with abundant clothing.

The clinical data of the individual patients included the diagnosis and history of the underlying neurological disease, e.g., PD, any coexisting postural abnormality, the reported angle of antecollis and the measurement method used in the original report, if available.

### Measurement of Postural Angles

Two independent raters (KA Woo, JY Joo) measured the upper camptocormia angle and the neck flexion angles in the figures. We used the freeware program GIMP 2.8.22, or the CamptoApp (@Spencer Kimbal, Peter Mattis et al.) to draw the lines and measure the postural angles [4].

The upper camptocormia angle was measured according to the 2018 consensus-based method [4]. One line was drawn from the L5 spinous process (L5) to the thoracic fulcrum, and another line from the fulcrum to the C7 spinous process (C7). The outer angle between the two lines was measured.

**Figure 1:**
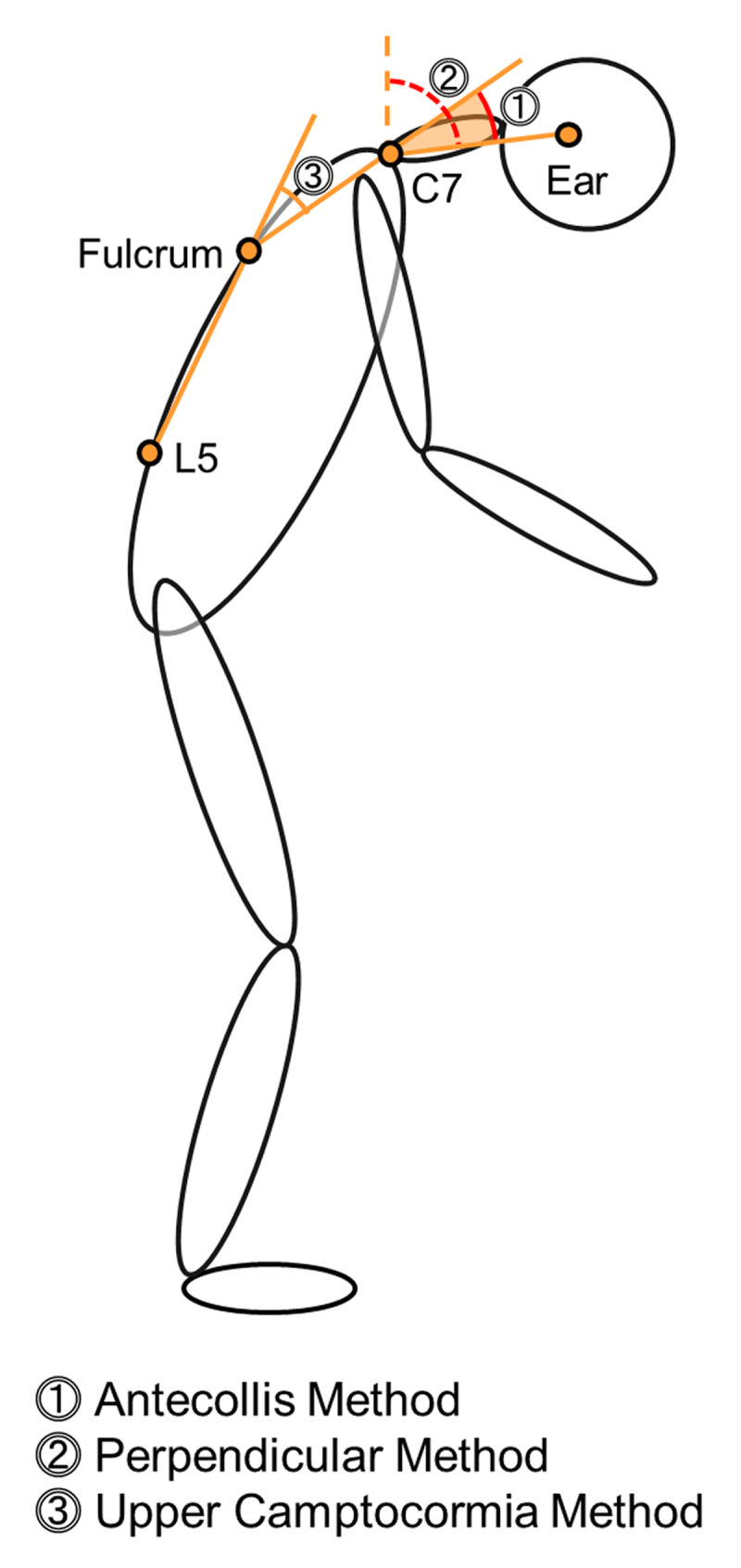
**Measurement methods of neck flexion angles and upper camptocormia angle in the patient photographs.** Postural angles were measured in the published photographs of antecollis patients in the literature. The figure depicts the measurement methods including the antecollis method and the perpendicular method for neck flexion, and the upper camptocormia method.

The neck flexion angles were measured with two different methods (Figure 1). The ‘perpendicular method’ measures the angle between the vertical line and the line drawn from the C7 spinous process to the ear tragus [5,6]. Since this method, as well as the wall goniometer, measures the angle in relation to the vertical line, the neck flexion angle is altered by truncal flexion. Thus, we developed the ‘antecollis method’ as a second measurement method to assess neck flexion in relation to the torso: the antecollis angle is defined as the angle between the fulcrum/C7 line and the C7/tragus line.

To assess interrater reliability and concordance for each method, absolute-agreement intraclass correlation coefficients (ICC) of the angles measured by two raters were statistically calculated using SPSS 23.0 (SPSS Inc, Chicago IL).

## Results

**Supplementary Figure 1:**
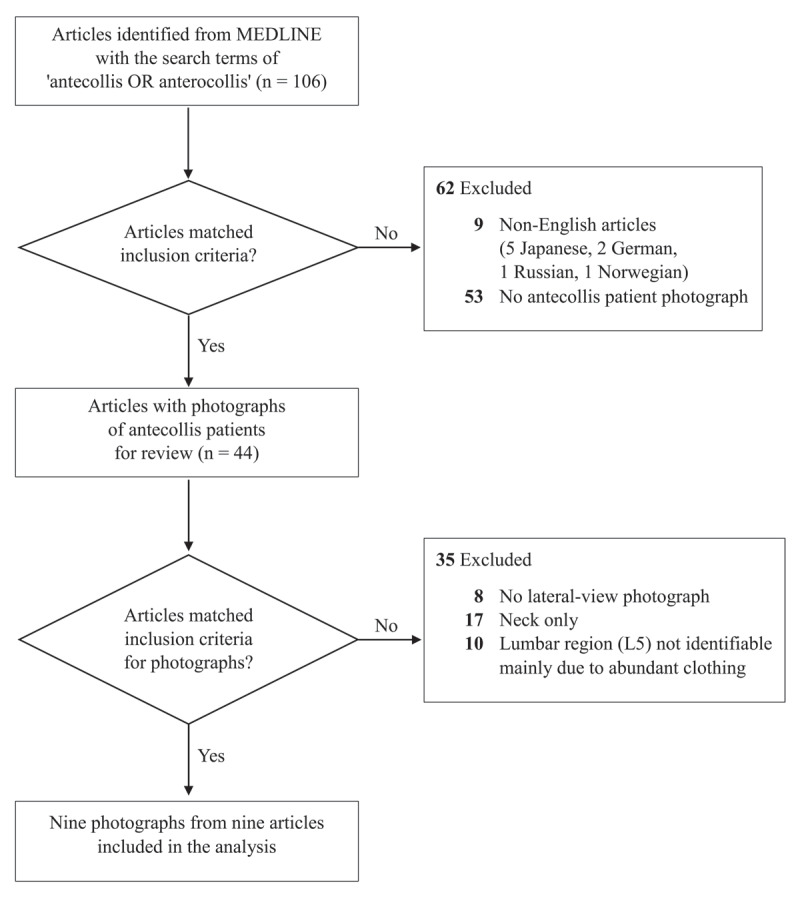
Flowchart of the Literature Review Process.

We identified nine patient photographs in nine studies meeting our inclusion criteria [6,7,8,9,10,11,12,13,14]. These publications included one case report, two case series, two clinical research on postural abnormalities, and review articles. The process of literature review is presented as a flowchart in the Supplementary Figure 1.

### Clinical Data of the Patients

Antecollis was the only described postural abnormality in eight cases, except for patient F who was reported to have antecollis, camptocormia, and Pisa syndrome (Table 1). Clinically, all of the nine patients exhibited parkinsonism. Seven were diagnosed with PD, one with probable MSA, and one with Dravet syndrome with *SCN1A* mutation. The angle of neck flexion was presented in only four cases, ranging from 54.41° to 95.0°.

**Table 1:** Clinical Information and Postural Angle Measurements of the Included Cases.


CASE	AUTHOR (YEAR)	CLINICAL DIAGNOSIS	ORIGINALLY DESCRIBED POSTURAL ABNORMALITY	NECK FLEXION ANGLE				UPPER CC ANGLE†

				ORIGINALLY REPORTED	ORIGINAL METHOD	PERPENDICULAR METHOD†	ANTECOLLIS METHOD†	

A	Tinazzi *et al*. (2019) [5]	PD	Antecollis	95°	PerpendicularMethod	94.7°	40.7°	61.9°

B	Tinazzi *et al*. (2019) [6]	PD	Antecollis	80°	Wall Goniometer	113.5°	56.2°	54.1°

C	Ruttiman *et al*. (2018) [7]	PD	Antecollis	–	–	94.25°	37.4°	71.8°

D	Ha *et al*. (2015) [8]	PD	Antecollis	–	–	101.65°	51.4°	60.05°

E	Kataoka *et al*. (2017) [9]	PD	Antecollis	54.41°	Vertical line, C7/vertex line	86.35°	40.7°	45.45°

F	Fasano *et al*. (2014) [10]	Dravet syndrome with *SCN1A* missense mutation	Antecollis, Camptocormia, Pisa syndrome	85°	Not specified	103.4°	51.05°	43.75°

G	Jankovic (2009) [11]	PD	Antecollis	–	–	113.75°	53.2°	56.95°

H	Suzuki *et al*. (2008) [12]	PD	Antecollisinduced by pramipexole	–	–	127.95°	61.3°	54.9°

I	Quinn(1989) [13]	ParkinsonismProbable MSA	Antecollis	–	–	97.4°	50.9°	58.7°


Abbreviations: *PD* Parkinson’s disease, *MSA* multiple system atrophy, *CC* camptocormia. † The average of the angles measured by two raters is presented.

Three articles demonstrated how neck flexion angle was measured: one measured the angle between the vertical line and the C7/vertex line, and the other two used vertically placed wall goniometers, with one additionally measuring the angle between the vertical line and the C7/tragus line which is consistent with the perpendicular method in our measurement [6,7,10].

### Neck Flexion and Upper Camptocormia Angles

The neck flexion angles measured with the perpendicular method exceeded 45° in all patients, with the mean angle of 103.7° (Table 1). Meanwhile, the angles measured with the antecollis method ranged from 37.4° to 61.3°, and the mean angle was 49.2°. The mean difference between the two neck flexion angles measured by the perpendicular method and the antecollis method was 54.5°.

The upper camptocormia angle measurement yielded the mean angle of 56.4°, ranging from 43.75° to 71.8°. Eight of the nine cases fulfilled the 45° criteria for upper camptocormia, except for patient F.

The ICC evaluating the absolute agreement between the two raters was 0.972 for the antecollis method, and 0.966 for the perpendicular method.

## Discussion

In the photographs of patients diagnosed with antecollis in the literature, we found increased upper camptocormia angle of 45° or larger in eight out of nine cases. The results suggest that upper camptocormia should be considered in the evaluation of antecollis. However, camptocormia was mentioned in only one of the nine original descriptions of these patients. In all three reports which specified the method of measurement for antecollis, the neck flexion angle was determined based on the vertical line which does not account for upper camptocormia thus inflating the degree of antecollis.

The antecollis method is based on the concept that the angle of neck flexion should be measured in relation to the torso rather than the vertical line. Despite the growing list of studies in the field of postural abnormalities, there has been no consensus on how to measure the angle of neck flexion. Three different methods were identified in the literature review: the perpendicular method, the wall goniometer, and the C7/vertex method [5,6,7,10]. These vertical line-based approaches are heavily influenced by lower truncal flexure. For instance, the angle of neck flexion by the perpendicular method is equivalent to the sum of (a) the upper camptocormia angle, (b) the angle between the vertical line and the L5/fulcrum line, and (c) the neck flexion angle by the antecollis method we propose (Figure 1). Therefore, this approach does not distinguish between antecollis and upper camptocormia in quantitative manners.

The antecollis method we propose utilizes the fulcrum/C7 line instead of vertical line. The fulcrum/C7 line is chosen because it is the line used to define the degree of upper truncal tilt in the consensus-based upper camptocormia method [4]. In our investigation of nine patient photographs, the neck flexion angle measured by the antecollis method was on average 54.5° smaller than the angle given by the perpendicular method. This discrepancy should be interpreted in light of the high prevalence of increased upper camptocormia angle in these patients, and we suggest that the antecollis method more accurately reflects the true degree of antecollis by excluding the contributions of upper truncal tilt. While one previous study used the line connecting the greater trochanter and the acromion to measure the ‘dropped head angle’ [15], the fulcrum/C7 line still has advantages in that greater trochanter is difficult to identify in the lateral view photographs of clothed patient, and that the trochanter/acromion line does not effectively reflect flexion limited to the thoracic area compared to the fulcrum/C7 line.

In our review of the literature, the photographs of antecollis patients varied greatly in terms of the angle, body regions included, and patient clothing. This calls for a standardized photograph protocol for the evaluation of antecollis. We suggest that the diagnosis of antecollis should be based on (a) lateral-view patient photographs including the (b) the entire upper body including the ear, C7, and L5 spinous processes, (c) taken with minimal clothing that reveals the silhouette of the back. Moreover, while the antecollis method consistently provided smaller neck flexion angles than the perpendicular method in our parkinsonian patients with forward stooped postures, we assume that the results may differ in other groups of patients e.g., those with exaggerated truncal lordosis. For patients with complex postural abnormalities, taking photographs from both sides of the torso may help to more accurately assess the effect of accompanying lateral flexion or rotation on the antecollis angle.

Some limitations of this study should be addressed. Several researchers also claim that antecollis can be further subdivided into conceptural antecollis and anterocaput, which respectively represents exclusive forward flexion of the cervical spine and the head [16]. This study did not address this classification, but we speculate that the proposed antecollis angle can be used to evaluate conceptual antecollis. While there are no guidelines for whether and how to measure the anterocaput angle, if a discussion is to take place, photographs taken according to the aforementioned protocol may help define the additional anatomical points required to assess anterocaput and various other postural abnormalities. In addition, considering that multiple neurological conditions may present with antercollis or anterocaput with or without camptocormia aside from MSA and PD, e.g. amyotrophic lateral sclerosis, myasthenia gravis, myopathy, and other neuromuscular disorders [17], validation in a larger, prospective cohort in these varied groups of patients is required. While the anatomical points including the C7 and L5 spinous processes were estimated in photographs of clothed patients, utilization of upper body radiographs in these larger groups of patients would facilitate a more robust analysis of the postural angles.
